# Gugulipid causes hypercholesterolemia leading to endothelial dysfunction, increased atherosclerosis, and premature death by ischemic heart disease in male mice

**DOI:** 10.1371/journal.pone.0184280

**Published:** 2017-09-14

**Authors:** Andrea Leiva, Susana Contreras-Duarte, Ludwig Amigo, Esteban Sepúlveda, Mauricio Boric, Verónica Quiñones, Dolores Busso, Attilio Rigotti

**Affiliations:** 1 Cellular and Molecular Physiology Laboratory (CMPL), Division of Obstetrics and Gynaecology, School of Medicine, Pontificia Universidad Católica de Chile, Santiago, Chile; 2 Department of Gastroenterology, School of Medicine, Pontificia Universidad Católica de Chile, Santiago, Chile; 3 Department of Physiology, Faculty of Biological Sciences, Pontificia Universidad Católica de Chile, Santiago, Chile; 4 Department of Nutrition, Diabetes and Metabolism, Pontificia Universidad Católica de Chile, Santiago, Chile; 5 Center of Molecular Nutrition and Chronic Diseases, School of Medicine, Pontificia Universidad Católica de Chile, Santiago, Chile; Centro Cardiologico Monzino, ITALY

## Abstract

For proper cholesterol metabolism, normal expression and function of scavenger receptor class B type I (SR-BI), a high-density lipoprotein (HDL) receptor, is required. Among the factors that regulate overall cholesterol homeostasis and HDL metabolism, the nuclear farnesoid X receptor plays an important role. Guggulsterone, a bioactive compound present in the natural product gugulipid, is an antagonist of this receptor. This natural product is widely used globally as a natural lipid-lowering agent, although its anti-atherogenic cardiovascular benefit in animal models or humans is unknown. The aim of this study was to determine the effects of gugulipid on cholesterol homeostasis and development of mild and severe atherosclerosis in male mice. For this purpose, we evaluated the impact of gugulipid treatment on liver histology, plasma lipoprotein cholesterol, endothelial function, and development of atherosclerosis and/or ischemic heart disease in wild-type mice; apolipoprotein E knockout mice, a model of atherosclerosis without ischemic complications; and SR-B1 knockout and atherogenic–diet-fed apolipoprotein E hypomorphic (SR-BI KO/ApoER61^h/h^) mice, a model of lethal ischemic heart disease due to severe atherosclerosis. Gugulipid administration was associated with histological abnormalities in liver, increased alanine aminotransferase levels, lower hepatic SR-BI content, hypercholesterolemia due to increased HDL cholesterol levels, endothelial dysfunction, enhanced atherosclerosis, and accelerated death in animals with severe ischemic heart disease. In conclusion, our data show important adverse effects of gugulipid intake on HDL metabolism and atherosclerosis in male mice, suggesting potential and unknown deleterious effects on cardiovascular health in humans. In addition, these findings reemphasize the need for rigorous preclinical and clinical studies to provide guidance on the consumption of natural products and regulation of their use in the general population.

## Introduction

Cholesterol homeostasis is a highly regulated metabolic process, and disturbances in this system determine the development of common human diseases such as atherosclerosis [1,2]. The regulation of lipoprotein receptor expression has particular relevance for cholesterol metabolism [3]. The main cellular receptors for low-density (LDL) and high-density (HDL) lipoproteins are the LDL receptor (LDLR) and the scavenger receptor class B type I (SR-BI), respectively [4,5]. SR-BI is involved in reverse cholesterol transport, a process in which cholesterol is removed from peripheral tissues by HDL and then transported to the liver to be secreted into the bile and eliminated in feces [6–8]. In animal models, deficiency of LDLR or SR-BI correlates with accelerated development of atherosclerosis after feeding of an atherogenic diet [9,10].

**Fig 1:**
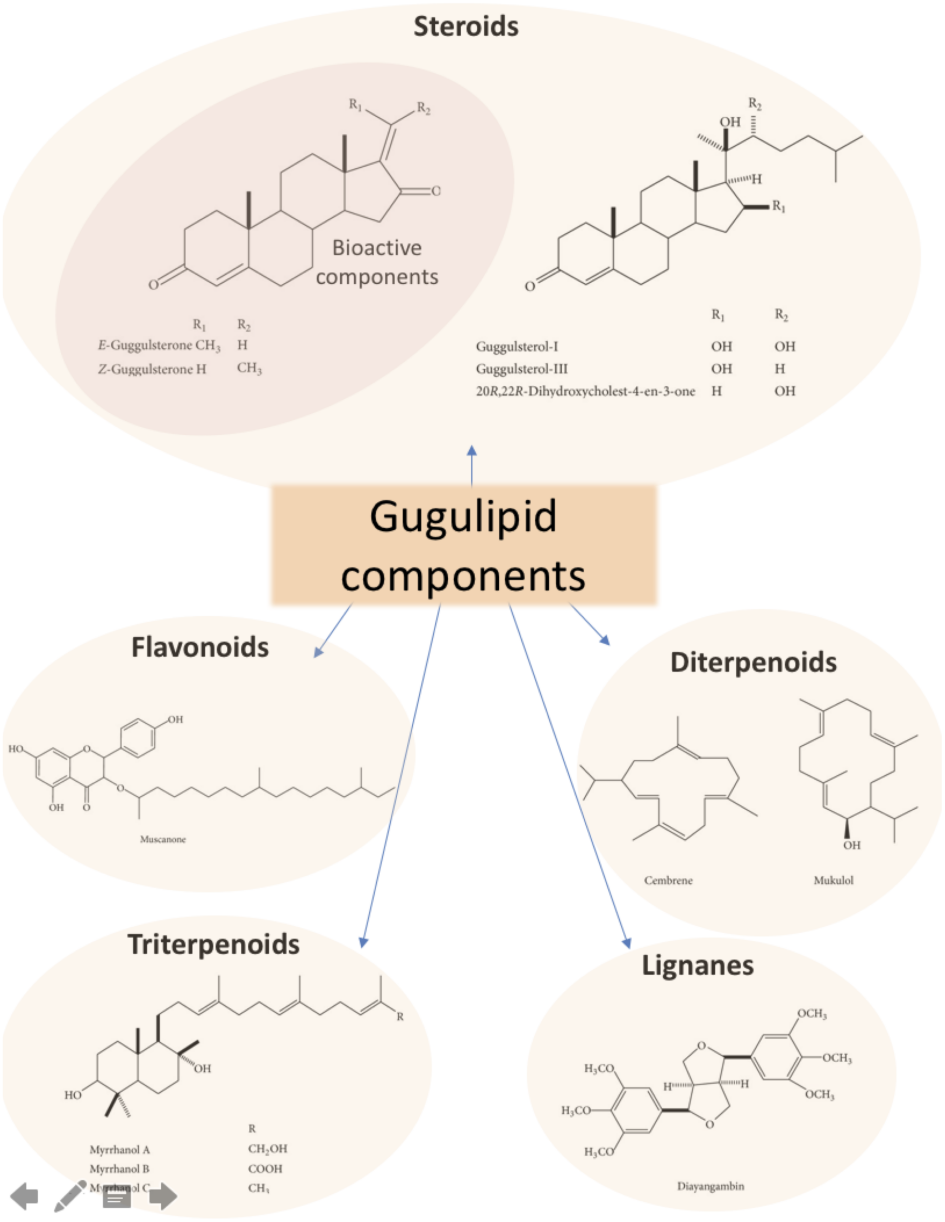
Principal components of gugulipid. The most studied bioactive compound present in guggul resin is gugulsterone (upper left panel). Other metabolites of the natural resin are also shown in this figure (based on references 16 and 21).

Gugulipid, made from gum resin of the *Commiphora mukul* tree, is a natural product widely used globally as a lipid-lowering agent, although its anti-atherogenic and cardiovascular benefits in humans or animal models are controversial [11–13]. Although studies published in India have reported its hypolipidemic properties, similar evidence in Western populations is still scarce and indicates that gugulipid consumption has neutral or deleterious effects on plasma cholesterol levels [11, 12]. The most relevant bioactive components present in gugulipid are the steroids guggulsterone E and Z although it also contains flavonoids, di- and triterpenoids, lignanes, long-chain aliphatic tetrols, aliphatic esters, ferulates, carbohydrates, and a variety of inorganic ions (Fig 1) [14, 15, 16].

Different studies have evaluated the presence of guggulsterone in various commercial gugulipid preparations, as well as its presence in blood samples after administration of this natural product [17–19]; however, gugulipid-related metabolic pathways have not been extensively analyzed. Some studies using guggulsterone—rather than total guggul resin—indicate that this key component can be transformed largely by CYP3A4 into more hydroxylated forms, which are more potent than guggulsterone itself in modulating gene expression in cultured liver cells [20]. In mice and rats, guggulsterones are antagonists of the nuclear farnesoid X receptor, thus favoring the hepatic catabolism of plasma cholesterol into bile acids [21, 22] and eventually leading to increased hepatic uptake of lipoprotein cholesterol and reduced plasma cholesterol levels.

Considering conflicting data on the impact of gugulipid on cholesterol homeostasis and that the effects of the natural extract may differ from those of pure guggulsterone, the aim of this study was to determine the effect of gugulipid on cholesterol homeostasis as well as to evaluate the physiological and pathophysiological impact of the treatment in mice. We studied the impact of gugulipid administration on lipoprotein cholesterol levels, endothelial function, and development of atherosclerosis and/or ischemic heart disease in male wild-type mice; apolipoprotein E knockout (ApoE KO) mice, a model of atherosclerosis without ischemic complications; and SR-BI knockout and atherogenic–diet-fed male apolipoprotein E hypomorphic (SR-BI KO/ApoER61^h/h^) mice, a model of lethal ischemic heart disease due to severe atherosclerosis.

## Materials and methods

### Animals and diets

C57BL/6 mice and apolipoprotein E knockout (ApoE KO) mice [23] were obtained from The Jackson Laboratory (Bar Harbor, ME, USA). SR-BI KO/ApoER61^h/h^ mice were obtained from Dr. Monty Krieger (Massachusetts Institute of Technology (MIT), Cambridge, MA, USA). Animals were housed with reverse light cycling under conditions of controlled temperature and humidity. Mice had free access to water and received standard low-fat/low-cholesterol chow diet (Prolab RMH3000; PMI Feeds Inc., St Louis, MO, USA) or an atherogenic diet (1.25% cholesterol, 15% total fat, and 0.5% cholic acid; 57BB; Test Diet, St Louis, MO, USA) to induce atherosclerotic cardiovascular disease [24] with or without gugulipid (Natrol, Chatsworth CA, USA or GNC, Pittsburgh, PA, USA) 5 mg/g diet for seven days or 1 mg/g diet for 12 weeks. These doses were determined based on the results of preliminary experiments that employed a range of concentrations (0.1–10 mg gugulipid/g diet) for 1 week and were lower than those used in previous studies [21]. Using 0.1–5 mg gugulipid/g diet), we observed no changes in food intake, body weight, or behavior. All experiments were performed in paired male littermates in control and treatment groups. At the beginning of the study, all mice were 2.5–3 months old and were followed up for 3 months or until death, whichever came first. Samples were obtained after overnight fasting. Protocols were approved by the Bioethics Committee of the School of Medicine, Pontificia Universidad Católica de Chile. Personnel involved in experiments described below were previously trained in animal handling and care by experienced research staff.

### Blood, liver, aorta, and heart sampling

Mice were anaesthetized with a mixture of ketamine:xylazine (150:10 mg/kg body weight). After anesthetization, blood was extracted, mice were euthanized, and the liver and heart were removed as described previously [25,26]. Plasma was prepared from blood by low-speed centrifugation and kept at -20°C. Livers were stored at -70°C until use. Aortas were removed under anesthesia for aortic ring preparation. Hearts used to determinate cardiomegaly and infarct scar size were excised and extracted and analyzed after spontaneous animal death. Hearts were frozen in cryopreservative solution and stored at -80°C until evaluation of atheromatous lesions.

### Hepatic histology

After liver removal, a small portion of each liver was fixed in neutral formalin followed by dehydration through a graded ethanol series and xylol, followed by embedding in paraffin at 60°C. Sections (7-μm) were prepared, mounted on glass slides, and rehydrated through a series of solutions of decreasing ethanol concentration. Finally, sections were stained with hematoxylin and eosin and observed under a light microscope.

### Plasma biochemical analysis

Commercial TGP/ALAT (Kovalent, Rio de Janeiro, Brazil) [27] and DuoSet ICAM-1 ELISA (R&D Systems, Minneapolis, MN, USA) [28] kits were used for plasma alanine aminotransferase (ALT) and soluble intercellular adhesion molecule-1 (ICAM) measurements, respectively.

### Cholesterol analysis

Plasma total cholesterol was enzymatically determined as previously described [26, 29–31]. Briefly, 10 μl of plasma or fast protein liquid chromatography (FPLC) fractions were incubated with 500 μl reaction solution (5 mM 4-clorophenol, 5 mM phenol, and 1% Triton X-100 in 0.5 M Tris-HCl [pH 7.6], 9 mM sodium cholate, 2 mM 4-aminoantipirine, 0.154 U/ml cholesterol esterase, 340 U/ml cholesterol oxidase, and 0.058 U/mg peroxidase) for 30 min at 37°C, and absorbance was measured at 550 nm. Cholesterol concentrations were calculated using a standard curve.

### Lipoprotein fractionation

Plasma was fractionated by gel filtration FPLC as previously described [30]. Briefly, plasma lipoproteins were separated on a Superose 6 column (Pharmacia, Uppsala, Sweden) installed in an FPLC device. Plasma (150 μl) was brought up to a volume of 300 μl with FPLC buffer (0.15 M NaCl, 1 mM EDTA, pH 7.4) and injected into the column. Chromatography was performed at room temperature with constant flux (0.3 ml/min) of FPLC buffer, and 40 fractions of 0.3 ml each were collected. Fractions were stored at -20°C until cholesterol analysis was performed.

### Isolation of total liver cell membranes

Hepatic tissue (0.1–0.15 g) was mixed with homogenization buffer (20 mM Tris [pH 7.4], 2 mM MgCl_2_, 0.25 M sucrose, and protease inhibitors) and lysed with an Ultra-turrax homogenizer (Kinematica, Lucerne, Switzerland). The homogenate was centrifuged at 3,000 *g* at 4°C for 10 minutes, and the resultant supernatant was centrifuged at 100,000 *g* at 4°C for 60 minutes. Pellets containing total hepatic membranes were resuspended in 100 μl homogenization buffer and stored at -20°C until analysis.

### Western blotting

Proteins from total hepatic total cell membranes were separated by SDS-PAGE and transferred to nitrocellulose membranes (BioRad Laboratories, Hercules, CA, USA) [29]. Immunoblotting was performed using a polyclonal rabbit anti–SR-BI antibody (1:3000 dilution) donated by Dr. Karen Kozarsky (SmithKline Beecham Pharmaceuticals, King of Prussia, PA, USA) [32] and an anti-LDLR antibody (1:2000 dilution) donated by Dr. Joachim Herz (University of Texas Southwestern Medical Center, Dallas, TX, USA). Polyclonal rabbit anti-ε-COP (1:5000 dilution) donated by Dr. Monty Krieger (MIT, Cambridge, MA, USA) [33] was used as a loading control. Membranes were then incubated with secondary horseradish peroxidase-conjugated anti-rabbit antibody (1:5000, Sigma-Aldrich, St Louis, MO, USA), and finally, protein/antibody complexes were detected by enhanced chemiluminescence and quantified by densitometry using Image J (National Institutes of Health, Bethesda, MD, USA).

### Vascular reactivity studies

Vascular reactivity was assessed as described elsewhere [34]. Briefly, the thoracic aorta was removed and ring segments of 2–4 mm were mounted in an organ bath system for isometric force measurements in Krebs physiological solution (120 mM NaCl, 2.7 mM CaCl_2_, 5.4 mM KCl, 1.2 mM MgCl_2_, 1.2 mM KH_2_PO_4_, 11 mM glucose, 10 mM Hepes, pH 7.4) maintained at 37°C and gassed with 5% CO_2_/95% air [34]. The optimal resting tension was determined with 50 mM KCl. Vessel rings were incubated in the presence or absence of phenylephrine (10^−9^ to 10^−5^ M), acetylcholine (10^−10^ to 10^−5^ M) and sodium nitroprusside (10^−10^ to 10^−5^ M). Tissue responses were expressed as a percentage of maximal contraction induced by 10^−6^ M phenylephrine.

### Recombinant adenovirus and infection

Adenovirus encoding wild-type murine SR-BI (Ad.SR-BI) and control virus without a transgene (Ad.E1Δ) were used. Large-scale production of recombinant adenoviruses was performed using infected HEK293 cells, and virus was purified by ultracentrifugation in a CsCl gradient. On day 0, C57BL/6 mice were injected via the femoral vein with phosphate-buffered saline or 1×10^11^ Ad.E1Δ particles (control) or with 1×10^11^ Ad.SR-BI particles (experimental). Plasma and liver samples were obtained on day 3 after adenoviral infection [29, 35, 36].

### Atherosclerotic lesion assessment

Atherosclerotic lesion size was determined and quantified in the aortic root as described previously [37–39]. Briefly, hearts were frozen in Tissue-Tek OCT (Sakura, Torrance, CA, USA). Serial 10-μm cross-sections (20 from each sample) through aortic roots were stained with Oil Red O and Mayer’s hematoxylin (Sigma-Aldrich). Images were captured for morphometric analysis, and lesion size was estimated in several sections of the same heart in which the three aortic valves were visualized (4–6 serial sections). Oil Red O staining was quantified using Image J software.

### Survival analysis

Lifespan was determined in SR-BI KO/ApoER61^h/h^ mice fed a standard chow diet (control), atherogenic diet, or atherogenic diet supplemented with gugulipid (1 mg/g diet) for 12 weeks. Feeding the atherogenic diet to this mouse strain leads to ischemic heart disease and sudden cardiovascular death—most likely due to severe cardiac arrhythmia—without major premonitory disease warnings [24,38]. Animals were visually inspected twice daily to assess health status. The endpoint for this experiment was defined as death or significant illness (based on poor general appearance [e.g., ruffled fur, abnormal gait], reduced food and water intake, decreased body weight, reduced activity). Animals reaching these endpoint criteria were immediately euthanized (n = 3); however most of the animals (13/16, 80% in groups fed an atherogenic diet with or without gugulipid supplementation) died suddenly before getting to the point of requiring euthanasia. The survival rate was determined by tabulating death of mice as a function of time in Kaplan-Meier curves as previously described [38].

### Statistical analysis

Results are shown as mean ± standard deviation (SD) or standard error of the mean (SEM), where n indicates the sample size (4–5 mice per group). The normality of the data was determined with the Kolmogorov-Smirnov test. Comparisons between two groups were performed using Student’s unpaired *t*-test or Mann-Whitney test for parametric or nonparametric data, respectively. Statistical significance of differences among more than two groups was determined by analysis of variance (ANOVA) or Friedman test for parametric or nonparametric data, respectively. If ANOVA or Friedman test demonstrated a significant interaction between variables, *post hoc* analyses were carried out by multiple-comparison Bonferroni or Dunn's correction tests, repectively. GraphPad Prism 6.0 (GraphPad Software, La Jolla, CA, USA) was used for data analysis. *P*<0.05 was considered statistically significant.

## Results

### Gugulipid administration leads to hepatic damage, hypercholesterolemia, and endothelial dysfunction

#### Liver histology and serum ALT levels

**Fig 2:**
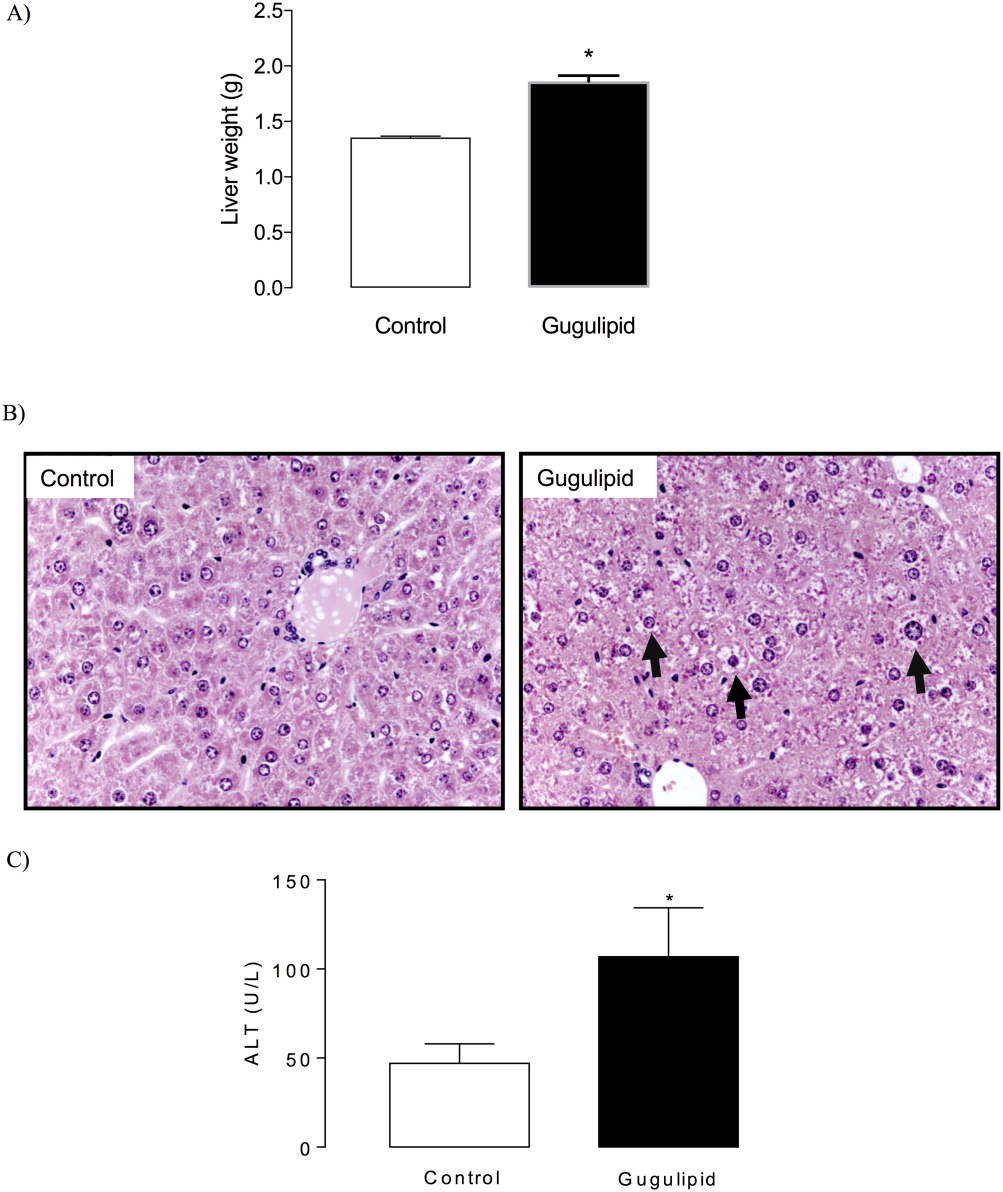
Effect of gugulipid on liver weight and histology and alanine transferase (ALT) level in male C57BL/6 mice. (A) Liver weight, (B) hepatic histology, and (C) plasma ALT level in control and gugulipid-treated mice. Arrows in (B) indicate enlarged hepatocytes with cytoplasmic tumefaction and vacuolization. Two independent experimental sets were used; n = 6 in each set. Values represent mean ± SD. *P<0.05.

In order to determine the effect of gugulipid on cholesterol metabolism, male C57BL/6 mice were fed a chow diet supplemented with 5 mg gugulipid/g diet for 7 days. At the end of treatment, the body weight of gugulipid-fed mice was similar to that of control mice (22.6 ± 0.6 vs. 23.6 ± 1.3 g, respectively). However, mice fed with gugulipid developed hepatomegaly, as indicated by increased liver weight (Fig 2A). Additionally, hepatic histology showed enlarged hepatocytes with cytoplasmic tumefaction and vacuolization (Fig 2B). Consistent with a significant degree of liver damage, plasma ALT levels were increased by 2-fold in gugulipid-treated mice (Fig 2C).

#### Plasma total and lipoprotein cholesterol and hepatic SR-BI protein levels

**Fig 3:**
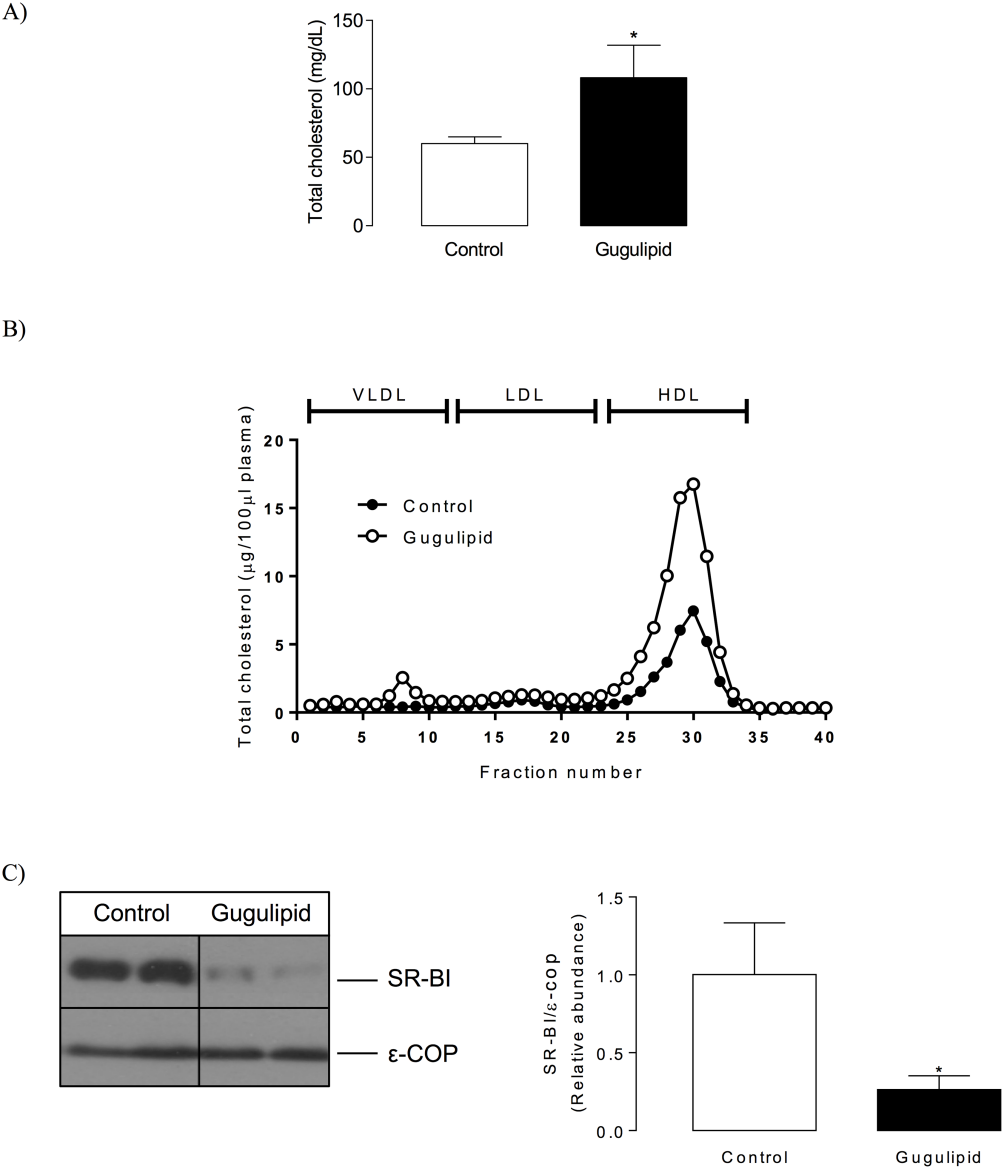
Effect of gugulipid on total and lipoprotein cholesterol levels and hepatic SR-BI level in male C57BL/6 mice. (A) Plasma total cholesterol level, (B) lipoprotein cholesterol distribution, and (C) western blot analysis (left) and quantitation (right) of hepatic SR-BI in control and gugulipid-treated mice. VLDL, very low-density lipoprotein; LDL, low-density lipoprotein; HDL, high-density lipoprotein. ε-COP served as loading control. Two independent experimental sets were analyzed; n = 6 in each set. Values represent mean ± SD. *P<0.05.

The level of total plasma cholesterol increased by 1.8-fold in gugulipid-treated C57BL/6 mice compared with the control group (Fig 3A). This hypercholesterolemic effect was mainly associated with an increase in HDL cholesterol (from 39.9± 2.6 in controls to 78.1 ±5.4 mg/dL in gugulipid-fed animals, *P*<0.05) and slighter changes in very low-density lipoprotein (VLDL) cholesterol level (Fig 3B). Because changes were detected in HDL, hepatic expression of the HDL receptor SR-BI was evaluated in order to determine if increased HDL cholesterol levels were associated with variations in this lipoprotein receptor in the liver. Indeed, western blot analysis showed that gugulipid administration led to a reduced (by approximately 75%) level of hepatic SR-BI (Fig 3C).

#### Endothelial function

**Fig 4:**
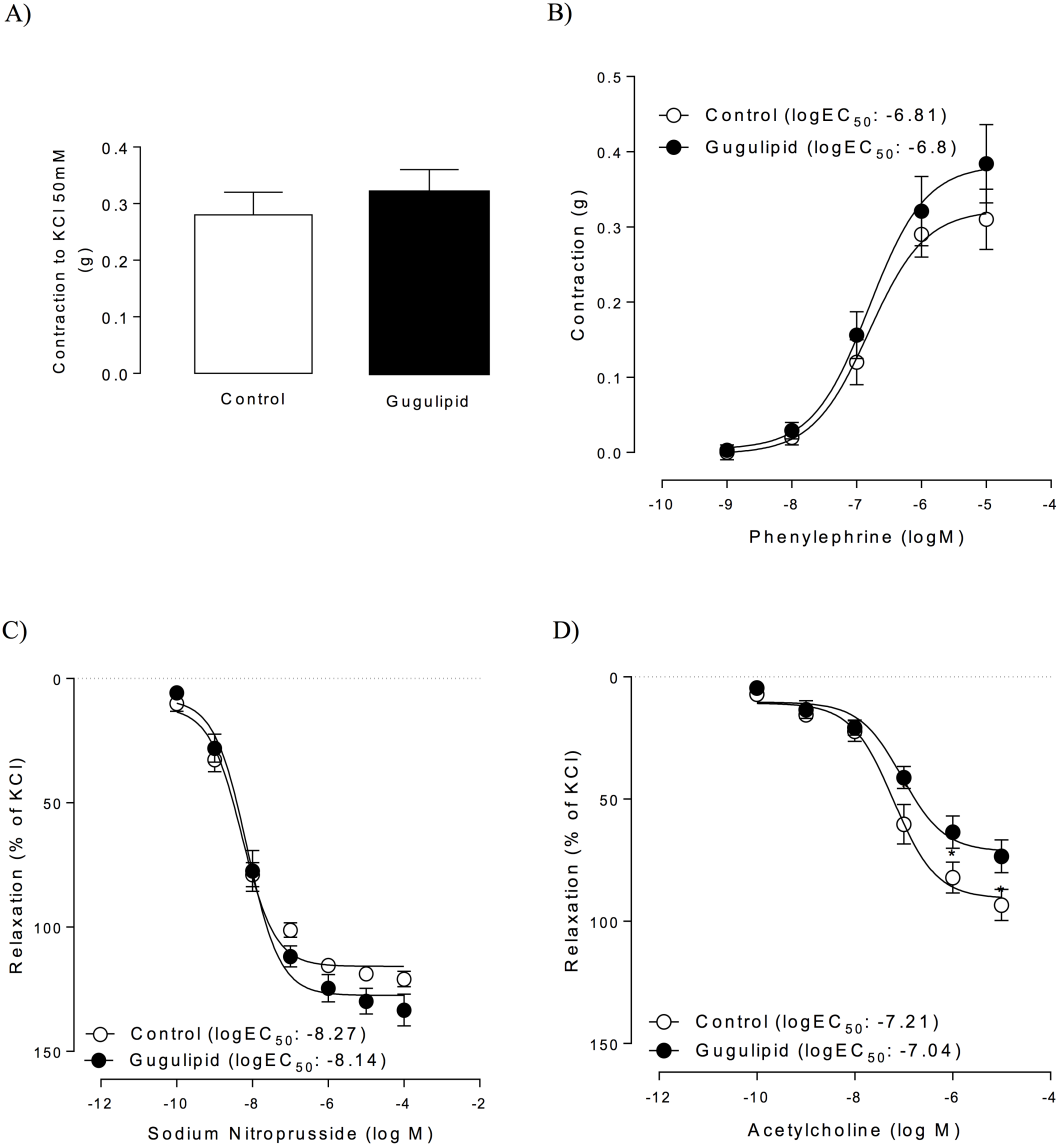
Effect of gugulipid on aortic vascular reactivity in male C57BL/6 mice. (A) Overall aortic contraction induced by KCl, (B) phenylephrine-induced vascular contraction, (C) sodium nitroprusside-induced relaxation, and (D) acetylcholine-induced relaxation in control and gugulipid-treated mice. In parenthesis is half-maximal effective dose (EC_50_) for each condition. Six independent experimental sets were used. In each experiment, vascular reactivity was assayed in parallel in one control and one gugulipid-treated mouse; n = 6 in each set. Values represent mean ± SD. *P<0.05.

Vascular reactivity was evaluated in aortic rings obtained from male C57BL/6 mice treated with gugulipid and control mice. Smooth–muscle-dependent contraction, induced by 50 mM KCl or by increasing concentrations of phenylephrine, did not differ between rings from gugulipid-treated and control mice (Fig 4A and 4B). Additionally, endothelium-independent relaxation induced by sodium nitroprusside was not affected by gugulipid administration (Fig 4C). In contrast, when the endothelium-dependent relaxation response was assayed with acetylcholine, the vasodilation response was reduced by 24% at a dose of 10^−6^ M and greater in aortic rings obtained from gugulipid-treated mice (Fig 4D). Furthermore, acetylcholine half-maximal effective concentration (*EC*_50_) was increased in aortas harvested from mice fed with gugulipid (Fig 4D).

### Gugulipid administration exacerbates hypercholesterolemia and endothelial damage, enhances atherosclerosis, and aggravates ischemic heart disease

#### Plasma and lipoprotein cholesterol levels in ApoE KO mice

**Fig 5:**
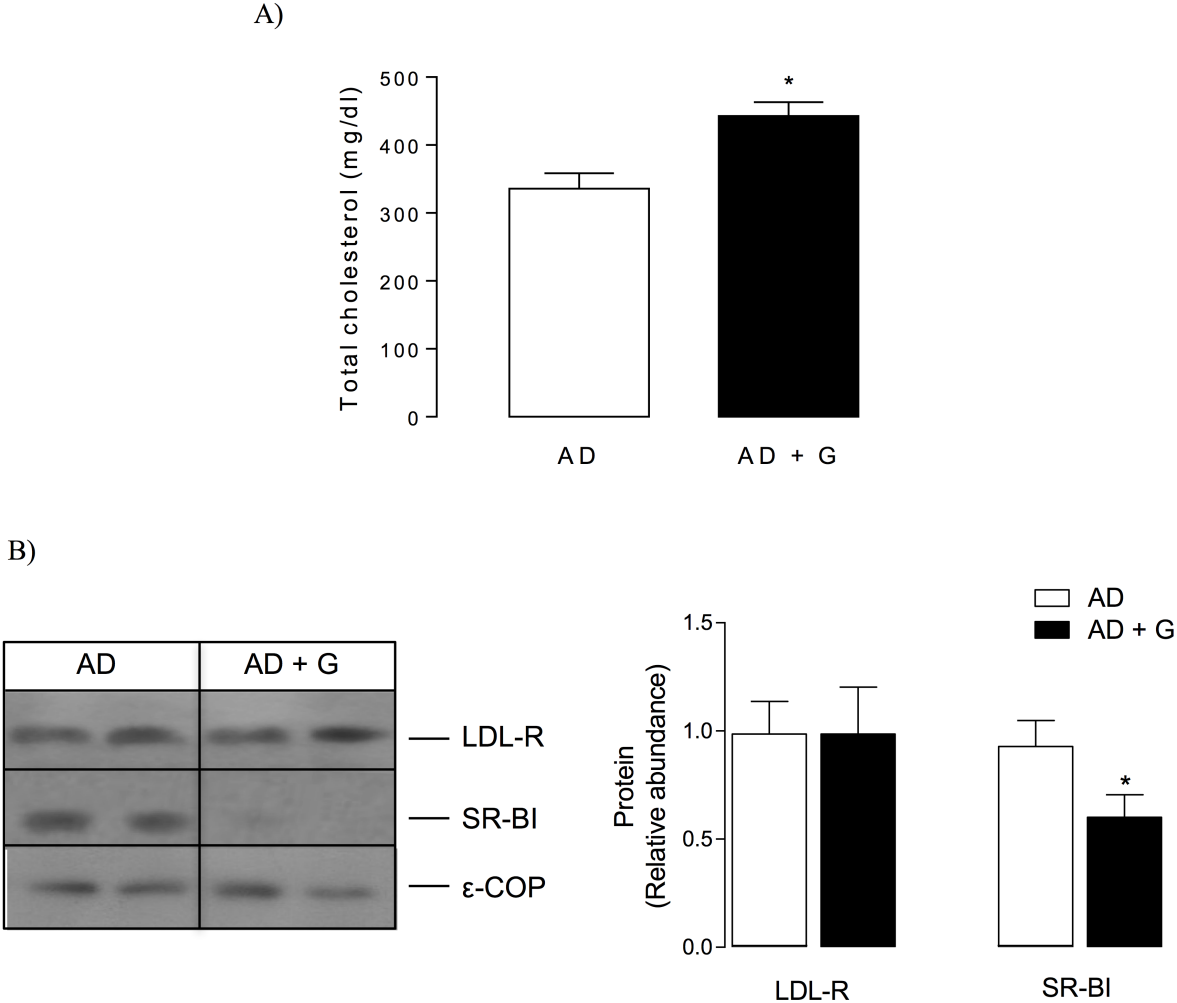
Effect of gugulipid on plasma cholesterol and hepatic SR-BI and LDL receptor (LDL-R) level in male ApoE KO mice. (A) Plasma total cholesterol and (B) western blot analysis (left) and quantitation (right) of hepatic LDL-R and SR-BI in control atherogenic diet-fed and gugulipid-treated atherogenic diet-fed mice. AD: atherogenic diet; G: gugulipid. Two independent experimental sets were used; n = 6 in each set. Values represent mean ± SD. *P<0.05.

The effect of gugulipid (1 mg/g of atherogenic diet by 12 weeks) on blood cholesterol level and progression of atherosclerosis was evaluated in atherogenic diet-fed male ApoE KO mice [39, 40]. In these mice, gugulipid caused a further increase in plasma total cholesterol level (Fig 5A), which was again associated with reduced hepatic SR-BI level without changes in LDLR expression (Fig 5B).

**Fig 6:**
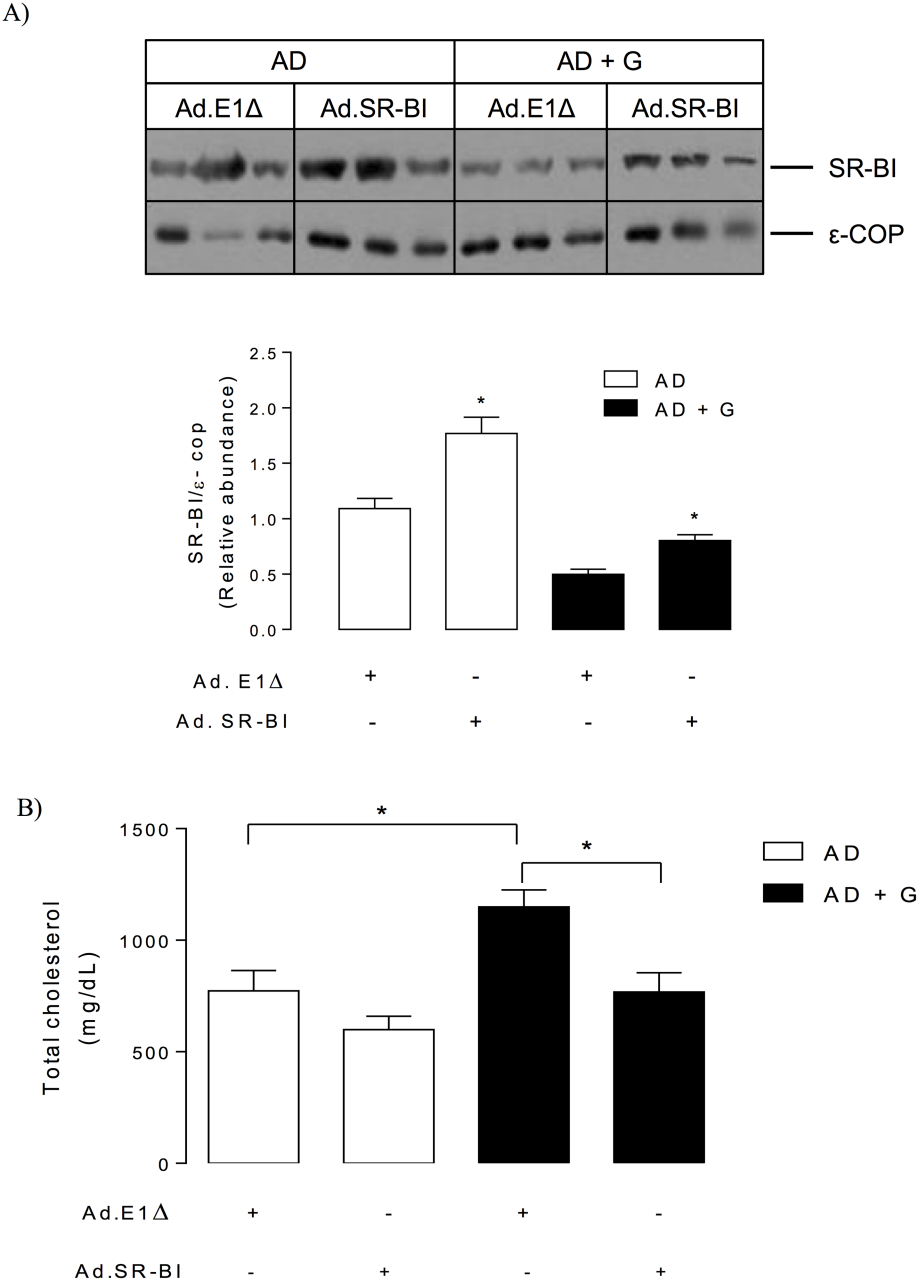
Effect of hepatic SR-BI overexpression on plasma cholesterol in gugulipid-treated atherogenic diet-fed male ApoE KO mice. (A) Western analysis (upper) and quantitation (lower) of hepatic SR-BI and (B) plasma total cholesterol levels in control atherogenic diet-fed and gugulipid-treated atherogenic diet-fed mice infected with adenovirus encoding murine SR-BI (Ad.SR-BI) or control adenovirus (Ad.E1Δ). AD: atherogenic diet; G: gugulipid. Two independent experimental sets were used, n = 6 in each. Values represent mean ± SD. *P<0.05.

In order to determine if reduced SR-BI expression was involved in the elevation of plasma cholesterol levels due to gugulipid treatment, male ApoE KO mice were infected with adenovirus encoding murine SR-BI (Ad.SR-BI) or control adenovirus (Ad.E1Δ) during the last week of feeding of the atherogenic diet with and without gugulipid. Hepatic SR-BI protein level was increased in ApoE KO mice infected with Ad.SR-BI after feeding of a control or gugulipid-supplemented diet (Fig 6A). Adenovirus-mediated hepatic SR-BI overexpression in gugulipid-treated ApoE KO mice reduced plasma total cholesterol to levels comparable to those observed in mice not treated with this natural product (Fig 6B).

#### Endothelial damage and atherosclerosis in atherogenic–diet-fed ApoE KO mice

**Fig 7:**
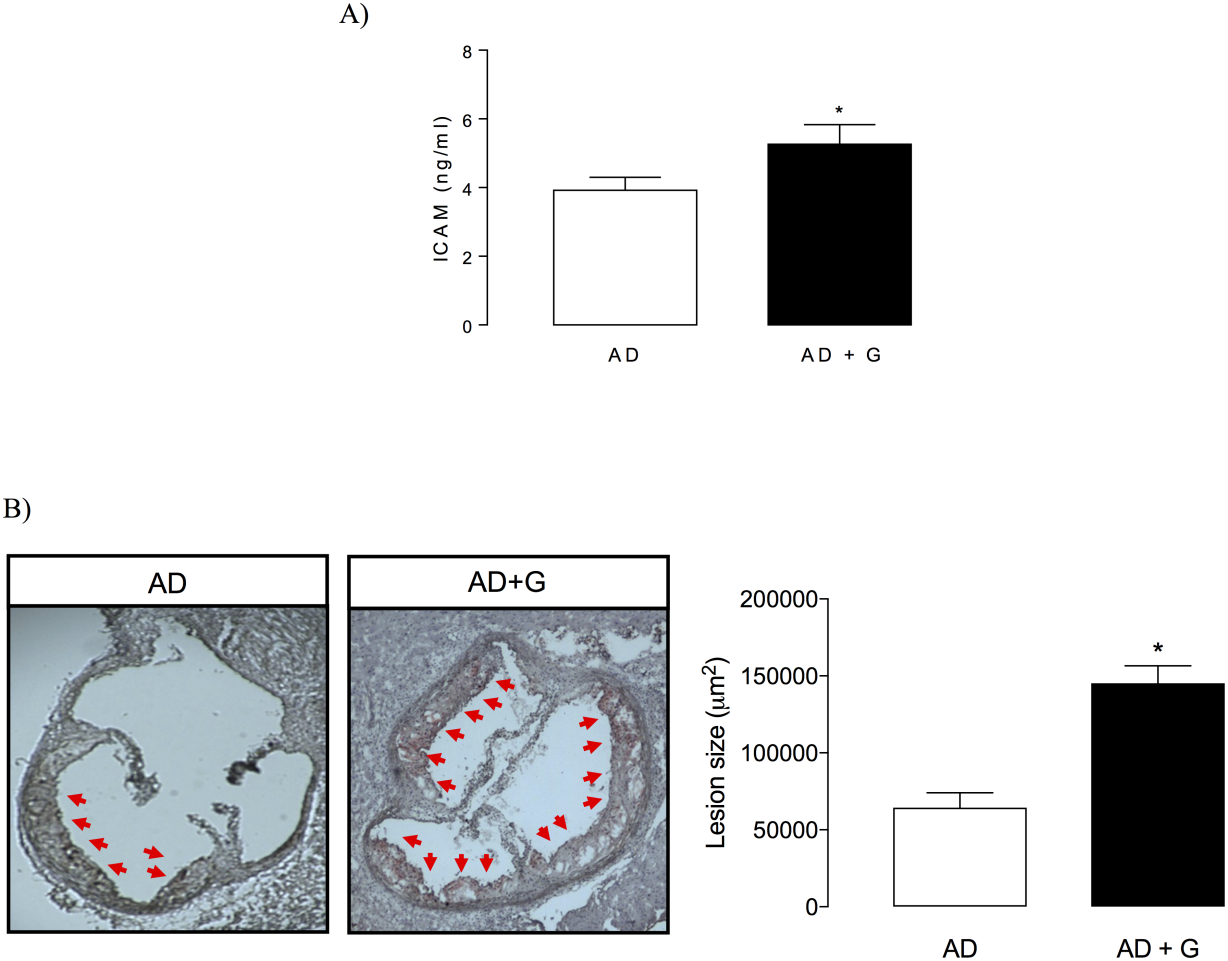
Effect of gugulipid on plasma intercellular adhesion molecule (ICAM) levels and atherosclerosis in atherogenic diet fed male apoE KO mice. (A) Plasma ICAM levels and (B) neutral lipid staining (left) and quantitation (right) of atheromatous lesion size in aortic roots of control and gugulipid-treated atherogenic diet-fed mice. Arrows indicate atheromatous lesions. AD: atherogenic diet; G: gugulipid. Two independent experimental sets were used, n = 6 in each. Values represent mean ± SD. *P<0.05.

Plasma levels of the soluble endothelial damage marker ICAM [28,41,42] were elevated in atherogenic diet-fed male ApoE KO mice treated with gugulipid compared with control mice (Fig 7A). Furthermore, aortic atherosclerotic lesion size was significantly increased by 2.5–3-fold after gugulipid administration in atherogenic–diet-fed ApoE KO mice (Fig 7B).

### Gugulipid administration aggravates ischemic heart disease and accelerates cardiovascular death in atherogenic–diet-fed male SR-BI KO/ApoER61^h/h^ mice

**Fig 8:**
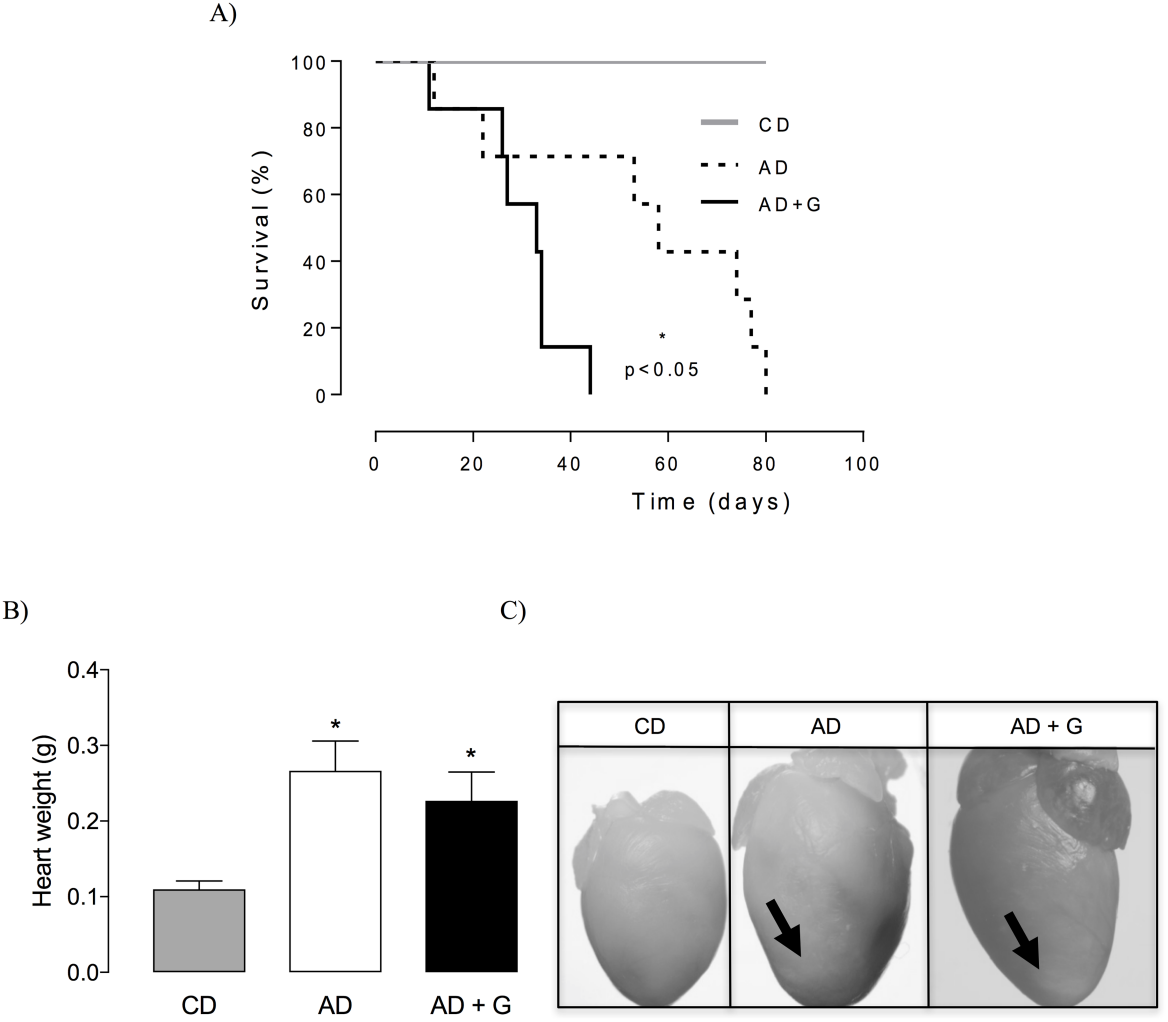
Effect of gugulipid on lifespan, heart size, and infarct scarring in atherogenic diet-fed male SR-BI KO/ApoER61^h/h^ mice. (A) Kaplan-Meier survival curve, (B) heart weight, and (C) heart infarct scarring (arrows) in control, atherogenic diet-fed, and gugulipid-treated atherogenic diet-fed mice. CD: control diet; AD: atherogenic diet; AD+G: atherogenic diet supplemented with gugulipid. Two independent experimental sets were used; n = 6–8 mice in each group. Values represent mean ± SD. *P<0.05.

Atherogenic–diet-fed SR-BI KO/ApoER61^h/h^ mice are a model of severe atherosclerotic coronary heart disease and ischemic complications leading to premature death [24]. Male mice fed a diet of 1 mg gugulipid/atherogenic diet were similar in overall body weight to mice fed a control or atherogenic diet (29.2 ± 2.9 g, 32.8 ± 2.9 g, and 30.1 ± 2.9 g, respectively). The administration of gugulipid showed that this treatment was associated with poorer survival (Fig 8A). Post-mortem analysis of hearts demonstrated significant cardiomegaly (Fig 8B) and larger myocardial infarction scars (Fig 8C) in animals fed gugulipid.

## Discussion

This study demonstrates significant deleterious effects of gugulipid treatment on liver tissue, HDL metabolism, and atherosclerosis in different mouse models, including wild-type and atherosclerosis-prone male mice.

First, gugulipid administration led to liver damage, including hepatomegaly, hepatocellular tumefaction, and elevated plasma ALT levels. In previous studies, guggulsterone induced apoptosis of liver cells [43] and abrogated the beneficial effects of some hepatoprotective agents in rodent models with liver damage [44–46]. In addition, the use of equisterol, an over-the-counter lipid-lowering product containing guggulsterone E and red yeast rice, was associated with hypertransaminasemia and severe hepatic lobular necro-inflammatory changes [47]. Due to lack of previous reports, the authors ruled out a role of guggulsterone as a cause of liver damage in this patient and they suggested that liver damage was due to the statin-like content of red yeast rice. Based on our study, potential hepatic adverse effects of gugulipid and/or guggulsterone should be considered during its clinical use. Indeed, toxicology studies of guggul, the gum resin obtained from the *Commiphora mukul* tree, have shown liver toxicity in patients with previous chronic inflammatory conditions [48]. Taken together, these findings advocate for more detailed side-effect studies on the use of guggul extract and its derivatives in clinical settings.

Initial data on the impact of gugulipid on lipid levels in humans from Asia indicated that this natural product had hypolipidemic effects, decreasing total cholesterol in normal subjects [49] and reducing total cholesterol and triglycerides and increasing HDL levels in dyslipidemic patients [50, 51]. In addition, gugulipid decreased total cholesterol, apparently as a consequence of increased LDL uptake and catabolism [52]. However, most of these studies were small and methodologically flawed [53]. Indeed, evidence of similar lipid-lowering in Western populations is lacking, and different studies suggest that gugulipid consumption has neutral or deleterious effects regarding plasma cholesterol levels [11,54].

With regard to plasma lipid levels, our study showed that gugulipid administration caused hypercholesterolemia rather than reducing total and LDL cholesterol levels or changing LDL receptor expression in male wild-type, as well as ApoE KO, mice. This hypercholesterolemic effect was due to increased levels of HDL cholesterol, which correlated with decreased hepatic expression of SR-BI, a well-established physiologically relevant HDL receptor [6–8]. To evaluate a potential role of SR-BI downregulation in determining increased HDL and total cholesterol levels in atherogenic–diet-fed male ApoE KO mice treated with gugulipid, overexpression of this receptor was achieved by recombinant adenoviral infection. Restoring hepatic SR-BI levels reduced plasma cholesterol levels, strongly suggesting the involvement of this receptor in the abnormal cholesterol metabolism seen in gugulipid-treated mice. Whereas many epidemiological and clinical studies indicate that increased HDL cholesterol levels are usually a marker of reduced cardiovascular risk [55], some high-HDL conditions, e.g., those due to reduced delivery of HDL cholesterol into the liver, may be associated with increased cardiovascular risk. Indeed, murine SR-BI deficiency, which induces high cholesterol levels in abnormal HDL particles and increased atherosclerosis under proatherogenic conditions, leads to a high rate of mortality from ischemic heart disease [8]. Furthermore, recent studies have established that anti-atherogenic functions of HDL, i.e., beyond its role in cholesterol metabolism may be as relevant as absolute HDL cholesterol level.

When vascular function was evaluated as an early indicator of cardiovascular disease, animals exposed to gugulipid showed altered endothelium-dependent reactivity but normal smooth muscle function, which suggested the presence of endothelial dysfunction. This latter finding was further supported by detection of increased levels of ICAM, a biomarker of endothelial dysfunction, in atherogenic–diet-fed male ApoE KO mice treated with gugulipid. To date, our study is the first report that links gugulipid use with endothelial dysfunction.

Evidence supporting an anti-atherogenic effect of gugulipid or guggulsterone is extremely limited. One study reported reduced atherogenesis in high-fat-and-cholesterol-fed albino rats treated with an ethylacetate extract obtained from guggul resin [56]. Our work is the first in which gugulipid was investigated in a more appropriate and established animal model of atherosclerosis to simultaneously evaluate its impact on plasma lipids, endothelial function, and atherogenesis. Based on its negative impact on SR-BI–dependent HDL metabolism and endothelial function, as well as a lack of cholesterol-lowering effect on non-HDL lipoproteins, gugulipid treatment appears to confer increased atherosclerotic risk. Indeed, gugulipid increased aortic lesion size in atherogenic–diet-fed male ApoE KO mice, a well-established animal model of accelerated atherosclerosis.

On the other hand, guggulsterone appears to be cardioprotective during isoproterenol-induced myocardial ischemia in rats [57]. In contrast to this previous report, our study showed a deleterious effect of gugulipid in a mouse model of severe atherosclerosis, myocardial infarction, and lethal ischemic heart disease [9,24]. Gugulipid reduced lifespan of atherogenic–diet-fed male SR-BI KO/ApoER61^h/h^ mice, which exhibited a greater degree of cardiomegaly and more extensive myocardial infarct scarring. These findings indicate that gugulipid accelerated atherosclerosis and/or increased ischemic complications in this model of severe coronary artery disease. Our work provides strong overall evidence supporting a pro-atherogenic effect of gugulipid in mice. Whether this deleterious effect is due to its guggulsterone content or is caused by some additional component present in this natural product remains to be established. However, our findings still have significant implications because gugulipid, but not guggulsterone, is the more commonly available and used formulation.

The discrepancies in effects reported after guggul administration in Eastern versus Western subjects, as well as in different animal models, may be explained by significant heterogeneity in gugulipid preparations, study design, and genetic and interspecies backgrounds. For instance, species- and population-dependent variation in metabolic pathways that transform guggulsterones and other guggul components (Fig 1) may contribute significantly to the dissimilar, and sometimes opposite, effects of guggul on blood lipids. In addition, guggul is usually consumed as a natural product rather than as purified guggulsterones. Thus, additional components in gugulipid (Fig 1) may also modulate the hypolipidemic activity of guggulsterone itself.

In summary, gugulipid impaired SR-BI–mediated HDL metabolism, caused endothelial dysfunction and liver damage, increased atherosclerosis, and accelerated cardiovascular death in male mice. Taken together, these data clearly show significant adverse effects of gugulipid administration and strongly suggest that the use of this natural product may have deleterious cardiovascular consequences for human health, in particular for patients at high risk for cardiovascular disease. Our findings are fully consistent with a recent review by the Natural Standard Research Collaboration group indicating that there is not enough scientific evidence to support the use of guggul for any medical condition and that it may cause serious side effects and interactions [53]. This study also highlights the need for rigorous preclinical and clinical studies to provide guidance on the consumption of natural products and regulation of their use in the general population.

## Supporting information

S1 File PLOS ONE humane endpoints checklist. (DOCX)

## References

[1] Maxfield FR, Tabas I. Role of cholesterol and lipid organization in disease. Nature. 2005;438:612–621. doi: 10.1038/nature04399 16319881 10.1038/nature04399

[2] Buckley ML, Ramji DP. The influence of dysfunctional signaling and lipid homeostasis in mediating the inflammatory responses during atherosclerosis. Biochim Biophys Acta. 2015;1852:1498–1510. doi: 10.1016/j.bbadis.2015.04.011 25887161 10.1016/j.bbadis.2015.04.011

[3] Goldstein JL, Brown MS. A century of cholesterol and coronaries: from plaques to genes to statins. Cell. 2015;161:161–172. doi: 10.1016/j.cell.2015.01.036 25815993 10.1016/j.cell.2015.01.036 PMC4525717

[4] Brown MS, Goldstein JL. A receptor-mediated pathway for cholesterol homeostasis. Science. 1986;232:34–47. 3513311 10.1126/science.3513311

[5] Acton S, Rigotti A, Landschulz KT, Xu S, Hobbs HH, Krieger M. Identification of scavenger receptor SR-BI as a high density lipoprotein receptor. Science. 1996;271:518–520. 8560269 10.1126/science.271.5248.518

[6] Babitt J, Trigatti B, Rigotti A, Smart EJ, Anderson RG, Xu S, et al Murine SR-BI, a high density lipoprotein receptor that mediates selective lipid uptake, is N-glycosylated and fatty acylated and colocalizes with plasma membrane caveolae. J Biol Chem. 1997;272:13242–13249. 9148942 10.1074/jbc.272.20.13242

[7] Krieger M. Charting the fate of the "good cholesterol": identification and characterization of the high-density lipoprotein receptor SR-BI. Annu Rev Biochem. 1999;68:523–558. doi: 10.1146/annurev.biochem.68.1.523 10872459 10.1146/annurev.biochem.68.1.523

[8] Trigatti BL, Krieger M, Rigotti A. Influence of the HDL receptor SR-BI on lipoprotein metabolism and atherosclerosis. Arterioscler Thromb Vasc Biol. 2003;23:1732–1738. doi: 10.1161/01.ATV.0000091363.28501.84 12920050 10.1161/01.ATV.0000091363.28501.84

[9] Braun A, Rigotti A, Trigatti BL. Myocardial infarction following atherosclerosis in murine models. Curr Drug Targets. 2008;9:217–223. 18336240 10.2174/138945008783755566

[10] Leiva A, Verdejo H, Benítez ML, Martínez A, Busso D, Rigotti A. Mechanisms regulating hepatic SR-BI expression and their impact on HDL metabolism. Atherosclerosis. 2011; 217:299–307. doi: 10.1016/j.atherosclerosis.2011.05.036 21741044 10.1016/j.atherosclerosis.2011.05.036

[11] Szapary PO, Wolfe ML, Bloedon LT, Cucchiara AJ, DerMarderosian AH, Cirigliano MD, et al Guggulipid for the treatment of hypercholesterolemia: a randomized controlled trial. JAMA. 2003;290:765–772. doi: 10.1001/jama.290.6.765 12915429 10.1001/jama.290.6.765

[12] Claudel T, Staels B, Kuipers F. The Farnesoid X receptor: a molecular link between bile acid and lipid and glucose metabolism. Arterioscler Thromb Vasc Biol. 2005;25:2020–2030. doi: 10.1161/01.ATV.0000178994.21828.a7 16037564 10.1161/01.ATV.0000178994.21828.a7

[13] Hasani-Ranjbar S, Nayebi N, Moradi L, Mehri A, Larijani B, Abdollahi M. The efficacy and safety of herbal medicines used in the treatment of hyperlipidemia; a systematic review. Curr Pharm Des. 2010;16:2935–2947 20858178 10.2174/138161210793176464

[14] Urizar NL, Liverman AB, Dodds DT, Silva FV, Ordentlich P, Yan Y, et al A natural product that lowers cholesterol as an antagonist ligand for FXR. Science. 2002;296:1703–1706. doi: 10.1126/science.1072891 11988537 10.1126/science.1072891

[15] Shah R, Gulati V, Palombo EA. Pharmacological properties of guggulsterones, the major active components of gum guggul. Phytother Res. 2012;26:1594–1605. doi: 10.1002/ptr.4647 22388973 10.1002/ptr.4647

[16] Sarup P, Bala S, Kamboj S. Pharmacology and phytochemistry of oleo-gum resin of Commiphorawightii (Guggulu). Scientifica (Cairo). 2015;2015:138039. 26587309 10.1155/2015/138039 PMC4637499

[17] Sane R, Bhate V, Malkar V, Nayak V, Kothurkar R. Reversed-phase high performance liquid chromatography determination of guggulsterones from pharmaceutical preparation and from blood plasma. Indian Drugs. 1990;28:86–89.

[18] Jain S, Kumar V, Aina, Singh R. Development and validation of HPLC-UV method for the determination of E and Z guggulsterone in rat plasma: Application to plasma protein binding study. World J Pharm Res. 2014;3:1616–27.

[19] Sairkar PK, Sharma A, Shukla NP. Estimation of guggulsterone E and Z in the guggul-based commercial formulations using high-performance thin-layer chromatography. J Pharm Bioallied Sci. 2017;9:1–7. doi: 10.4103/0975-7406.206225 28584486 10.4103/0975-7406.206225 PMC5450464

[20] Yang D, Yang J, Shi D, Xiao D, Chen Y, Black C, Deng R, Yan B. Hypolipidemic agent Z-guggulsterone: metabolism interplays with induction of carboxylesterase and bile salt export pump. J Lipid Res. 2012; 53:529–539. doi: 10.1194/jlr.M014688 22246918 10.1194/jlr.M014688 PMC3276476

[21] Urizar NL, Moore DD. Gugulipid: a natural cholesterol-lowering agent. Annu Rev Nutr. 2003;23:303–313. doi: 10.1146/annurev.nutr.23.011702.073102 12626688 10.1146/annurev.nutr.23.011702.073102

[22] Singh V, Kaul S, Chander R, Kapoor NK. Stimulation of low density lipoprotein receptor activity in liver membrane of guggulsterone treated rats. Pharmacol Res. 1990;22:37–44. 10.1016/1043-6618(90)90741-u 2330337

[23] Zhang SH, Reddick RL, Piedrahita JA, Maeda N. Spontaneous hypercholesterolemia and arterial lesions in mice lacking apolipoprotein E. Science. 1992;258:468–471. 1411543 10.1126/science.1411543

[24] Zhang S, Picard MH, Vasile E, Zhu Y, Raffai RL, Weisgraber KH, et al Diet-induced occlusive coronary atherosclerosis, myocardial infarction, cardiac dysfunction, and premature death in scavenger receptor class B type I-deficient, hypomorphic apolipoprotein ER61 mice. Circulation. 2005;111:3457–3464. doi: 10.1161/CIRCULATIONAHA.104.523563 15967843 10.1161/CIRCULATIONAHA.104.523563

[25] Amigo L, Quiñones V, Mardones P, Zanlungo S, Miquel JF, Nervi F, et al Impaired biliary cholesterol secretion and decreased gallstone formation in apolipoprotein E-deficient mice fed a high-cholesterol diet. Gastroenterology. 2000;118:772–779. 10734029 10.1016/s0016-5085(00)70147-8

[26] Mardones P, Quiñones V, Amigo L, Moreno M, Miquel JF, Schwarz M, et al Hepatic cholesterol and bile acid metabolism and intestinal cholesterol absorption in scavenger receptor class B type I-deficient mice. J Lipid Res. 2001;42:170–180. 11181745

[27] Morales MG, Amigo L, Balboa E, Acuña M, Castro J, Molina H, et al Deficiency of Niemann-Pick C1 protein protects against diet-induced gallstone formation in mice. Liver Int. 2010;30:887–897. doi: 10.1111/j.1478-3231.2010.02230.x 20408952 10.1111/j.1478-3231.2010.02230.x

[28] Fisker Hag AM, Pedersen SF, Kjaer A. Gene expression of LOX-1, VCAM-1, and ICAM-1 in pre-atherosclerotic mice. Biochem Bioph Res Comm. 2008;377:689–693. 10.1016/j.bbrc.2008.10.037 18930708

[29] Mardones P, Pilon A, Bouly M, Duran D, Nishimoto T, Arai H, et al Fibrates down-regulate hepatic scavenger receptor class B type I protein expression in mice. J Biol Chem. 2003;278:7884–7890. doi: 10.1074/jbc.M211627200 12511553 10.1074/jbc.M211627200

[30] Rigotti A, Trigatti BL, Penman M, Rayburn H, Herz J, Krieger M. A targeted mutation in the murine gene encoding the high density lipoprotein (HDL) receptor scavenger receptor class B type I reveals its key role in HDL metabolism. Proc Natl Acad Sci USA. 1997;94:12610–12615. 9356497 10.1073/pnas.94.23.12610 PMC25055

[31] Allain CC, Poon LS, Chan CS, Richmond W, Fu PC. Enzymatic determination of total serum cholesterol. Clin Chem. 1974;20:470–475. 4818200

[32] Kozarsky K, Krieger M. Scavenger receptor class B, type I-mediated [3H]cholesterol efflux to high and low density lipoproteins is dependent on lipoprotein binding to the receptor. J Biol Chem 2000;275:29993–30001. 11001950 10.1074/jbc.275.39.29993

[33] Guo Q, Penman M, Trigatti BL, Krieger M. A single point mutation in epsilon-COP results in temperature-sensitive, lethal defects in membrane transport in a Chinese hamster ovary cell mutant. J Biol Chem. 1996;271:11191–11196. 8626666 10.1074/jbc.271.19.11191

[34] Russell A, Watts S. Vascular reactivity of isolated thoracic aorta of the C57BL/6J mouse. The Jo Pharm Exp Ther. 2000;294:598–604. 10900237

[35] Yesilaltay A, Morales MG, Amigo L, Zanlungo S, Rigotti A, Karackattu SL, et al Effects of hepatic expression of the high-density lipoprotein receptor SR-BI on lipoprotein metabolism and female fertility. Endocrinology. 2006; 147:1577–1588. doi: 10.1210/en.2005-1286 16410302 10.1210/en.2005-1286

[36] Kozarsky KF, Donahee MH, Rigotti A, Iqbal SN, Edelman ER, Krieger M. Overexpression of the HDL receptor SR-BI alters plasma HDL and bile cholesterol levels. Nature. 1997; 387:414–417. doi: 10.1038/387414a0 9163428 10.1038/387414a0

[37] Trigatti B, Rayburn H, Viñals M, Braun A, Miettinen H, Penman M, et al Influence of the high density lipoprotein receptor SR-BI on reproductive and cardiovascular pathophysiology. Proc Natl Acad Sci U S A. 1999;96:9322–9327. 10430941 10.1073/pnas.96.16.9322 PMC17781

[38] Braun A, Trigatti BL, Post MJ, Sato K, Simons M, Edelberg JM, et al Loss of SR-BI expression leads to the early onset of occlusive atherosclerotic coronary artery disease, spontaneous myocardial infarctions, severe cardiac dysfunction, and premature death in apolipoprotein E-deficient mice. Circ Res. 2002;90:270–276. 11861414 10.1161/hh0302.104462

[39] Braun A, Rigotti A, Trigatti BL. Myocardial infarction following atherosclerosis in murine models. Curr Drug Targets. 2008;9:217–223. 18336240 10.2174/138945008783755566

[40] Plump AS, Smith JD, Hayek T, Aalto-Setälä K, Walsh A, Verstuyft JG, et al Severe hypercholesterolemia and atherosclerosis in apolipoprotein E-deficient mice created by homologous recombination in ES cells. Cell. 1992;71:343–353. 1423598 10.1016/0092-8674(92)90362-g

[41] Blankenberg S, Barbaux S, Tiret L. Adhesion molecules and atherosclerosis. Atherosclerosis. 2003;170:191–203. 14612198 10.1016/s0021-9150(03)00097-2

[42] Seth R, Raymond FD, Makgoba MW. Circulating ICAM-1 isoforms: diagnostic prospects for inflammatory and immune disorders. Lancet. 1991;338:83–84. 1676471 10.1016/0140-6736(91)90077-3

[43] Moon DO, Park SY, Choi YH, Ahn JS, Kim GY. Guggulsterone sensitizes hepatoma cells to TRAIL-induced apoptosis through the induction of CHOP-dependent DR5: involvement of ROS-dependent ER-stress. Biochem Pharmacol. 2011;82:1641–1650. doi: 10.1016/j.bcp.2011.08.019 21903093 10.1016/j.bcp.2011.08.019

[44] Meng Q, Chen X, Wang C, Liu Q, Sun H, Sun P, et al Alisol B 23-acetate promotes liver regeneration in mice after partial hepatectomy via activating farnesoid X receptor. Biochem Pharmacol. 2014;92:289–298. doi: 10.1016/j.bcp.2014.09.009 25278094 10.1016/j.bcp.2014.09.009

[45] Xu W, Lu C, Yao L, Zhang F, Shao J, Zheng S. Dihydroartemisinin protects against alcoholic liver injury through alleviating hepatocyte steatosis in a farnesoid X receptor-dependent manner. Toxicol Appl Pharmacol. 2017;315:23–34. doi: 10.1016/j.taap.2016.12.001 27939985 10.1016/j.taap.2016.12.001

[46] Xu W, Lu C, Zhang F, Shao J, Yao S, Zheng S. Dihydroartemisinin counteracts fibrotic portal hypertension via farnesoid X receptor dependent inhibition of hepatic stellate cell contraction. FEBS J. 2017;284:114–133. doi: 10.1111/febs.13956 27896916 10.1111/febs.13956

[47] Grieco A, Miele L, Pompili M, Biolato M, Vecchio FM, Grattagliano I, et al Acute hepatitis caused by a natural lipid-lowering product: when "alternative" medicine is no "alternative" at all. J Hepatol. 2009;50:1273–1277. doi: 10.1016/j.jhep.2009.02.021 19398239 10.1016/j.jhep.2009.02.021

[48] Masten SA. Gum guggul and some of its steroidal constituents: Review of toxicological literature. US Department of Health and Human Services, National Toxicology Program (NTP), National Institute of Environmental Health Sciences (NIEHS), National Institutes of Health. 2005;2: 1–39.

[49] Ghorai M, Mandal SC, Pal M, Pal SP, Saha BP. A comparative study on hypocholesterolaemic effect of allicin, whole germinated seeds of bengal gram and guggulipid of gum guggul. Phytother Res. 2000;14:200–202. 10815015 10.1002/(sici)1099-1573(200005)14:3<200::aid-ptr548>3.0.co;2-t

[50] Nityanand S, Srivastava JS, Asthana OP. Clinical trials with gugulipid. A new hypolipidaemic agent. J Assoc Physicians India. 1989;37:323–328. 2693440

[51] Nohr LA, Rasmussen LB, Straand J. Resin from the mukul myrrh tree, guggul, can it be used for treating hypercholesterolemia? A randomized, controlled study. Complement Ther Med. 2009;17:16–22. 19114224 10.1016/j.ctim.2008.07.001

[52] Singh RB, Niaz MA, Ghosh S. Hypolipidemic and antioxidant effects of Commiphora mukul as an adjunct to dietary therapy in patients with hypercholesterolemia. Cardiovasc Drugs Ther. 1994;8:659–664. 7848901 10.1007/BF00877420

[53] Ulbricht C, Basch E, Szapary P, Hammerness P, Axentsev S, Boon H, et al Guggul for hyperlipidemia: a review by the Natural Standard Research Collaboration. Complement Ther Med. 2005;13:279–290 doi: 10.1016/j.ctim.2005.08.003 16338199 10.1016/j.ctim.2005.08.003

[54] Das Gupta R. Pro-lipidaemic effect. J Indian Med Assoc. 1990;88:346 2074357

[55] Gordon T, Castelli WP, Hjortland MC, Kannel WB, Dawber TR. High density lipoprotein as a protective factor against coronary heart disease. The Framingham Study. Am J Med. 1977;62:707–714. 193398 10.1016/0002-9343(77)90874-9

[56] Lata S, Saxena KK, Bhasin V, Saxena RS, Kumar A, Srivastava VK. Beneficial effects of Allium sativum, Allium cepa and Commiphora mukul on experimental hyperlipidemia and atherosclerosis: a comparative evaluation. J Postg Med. 1991, 27: 132–135. 1784023

[57] Chander R, Rizvi F, Khanna AK, Pratap R. Cardioprotective activity of synthetic guggulsterone (E and Z-isomers) in isoproterenol induced myocardial ischemia in rats: A comparative study. Indian J Clin Biochem. 2003;18:71–79. 10.1007/BF02867370 PMC3453889 23105395

